# Optimal timing of introducing mobilization therapy for ICU patients with sepsis

**DOI:** 10.1186/s40560-022-00613-8

**Published:** 2022-04-25

**Authors:** Keibun Liu, Junichiro Shibata, Kiyoyasu Fukuchi, Kunihiko Takahashi, Tomohiro Sonoo, Takayuki Ogura, Tadahiro Goto

**Affiliations:** 1 Critical Care Research Group, Faculty of Medicine, University of Queensland and The Prince Charles Hospital, 627 Rode Road, Chermside, Brisbane, QLD 4032 Australia; 2 Faculty of Medicine, The University of Tokyo, Tokyo, Japan; 3 Department of Biostatistics, M&D Data Science Center, Tokyo Medical and Dental University, Tokyo, Japan; 4 TXP Medical Co. Ltd., Tokyo, Japan; 5 Department of Emergency and Critical Care Medicine, Hitachi General Hospital, Hitachi, Ibaraki Japan; 6 Department of Emergency Medicine and Critical Care Medicine, Tochigi Prefectural Emergency and Critical Care Centre, Imperial Foundation Saiseikai Utsunomiya Hospital, Tochigi, Japan; 7 Department of Public Health, Graduate School of Medicine, The University of Tokyo, Tokyo, Japan

**Keywords:** Early mobilization, Muscle wasting, Sepsis, ICU, IPW

## Abstract

**Background:**

For patients admitted to the intensive care unit (ICU) with sepsis, mobilization therapy during ICU stay can improve their outcomes during and after the ICU stay. However, little is known about the optimal timing of introducing mobilization therapy.

**Methods:**

This is a retrospective cohort study using data from a tertiary medical center in Japan during 2013–2017. We included patients aged ≥ 18 years who were admitted to the ICU with sepsis based on the Sepsis-3 criteria. We defined early mobilization (EM) as the rehabilitation at the level of sitting on the edge of the bed or more within the first 3 days of the patients’ ICU stay. Patients were divided into the EM and non-EM groups. The primary outcomes were in-hospital mortality and ambulatory dependence at hospital discharge. We estimated the effects of EM by stabilized inverse probability weighting (sIPW). We then tested alternative definitions of EM by changing the cutoff in days to mobilization by 1-day increments from 2 to 7 days to investigate the optimal timing of mobilization.

**Results:**

Our study sample consisted of a total of 296 septic patients, including 96 patients in the EM group and 200 patients in the non-EM group. In the sIPW model, the adjusted OR for in-hospital mortality in the EM group compared to the non-EM group was 0.22 [95% CI 0.06–0.88], and the adjusted OR for ambulatory dependence at the hospital discharge was 0.24 [95% CI 0.09–0.61]. When alternative definitions of EM were tested, patients who achieved mobilization within the first 2–4 days of their ICU stays had better outcomes.

**Conclusions:**

Achieving mobilization within the first 3 days of ICU stay was significantly associated with better outcomes. Patients with sepsis might benefit most from achieving mobilization within 2–4 days. Further studies are warranted to validate the findings.

**Supplementary Information:**

The online version contains supplementary material available at 10.1186/s40560-022-00613-8.

## Background

For patients admitted to the intensive care unit (ICU), introducing mobilization therapy during ICU stay can improve physical, cognitive, and psychological functioning during and after the ICU stay and prevent post-intensive care syndrome (PICS) [1–7]. Since patients who develop PICS experience a significant decrease in their activity levels in daily life and could even die after hospital discharge, it is clinically and economically crucial to prevent the onset and progression of PICS [8–13].

Several previous studies have shown that mobilization therapy during ICU stay contributes to preventing PICS [14–22]. One retrospective cohort study of ICU patients with community-acquired pneumonia showed that ICU mobilization reduced patients’ in-hospital mortality [14]. Another prospective cohort study of mechanically ventilated patients showed that about 70% of the patients who received mobilization therapy during their ICU stays were able to maintain sufficient walking function after leaving the ICU [15].

The optimal timing of introducing mobilization therapy during an ICU stay, however, has not been adequately discussed, and the degree of improvement in patient outcomes has varied across studies [14–22]. For instance, a meta-analysis of 15 randomized controlled trials (RCTs) on mobilization interventions for mechanically-ventilated ICU patients indicated that the initiation of mobilization therapy within 48–72 h of mechanical ventilation may be optimal for improving the clinical outcome of patients [16]. Other studies have also supported the potential benefits of early initiation of mobilization, such as within 72 h, rather than late initiation [14, 15, 17]. By contrast, another study on patients during the first 7 days of their ICU stays found little reduction in in-hospital mortality or improvement in physical function after ICU discharge by mobilization therapy in the ICU [22]. The adequate timing to introduce mobilization therapy during ICU stays to maximize improvement in patient outcomes remains controversial.

Therefore, we hypothesized that early mobilization within the first 3 days of ICU admission would maximize improvement in patient outcomes. To test this hypothesis, we analyzed data from one of the largest tertiary hospitals in Japan to examine differences in outcomes according to the timing of achieving mobilization in patients with sepsis, an important risk factor for developing PICS in the ICU [23].

## Methods

### Study design and settings

This is a retrospective observational study using data from the ICU of the Japanese Red Cross Maebashi Hospital from July 2013 to June 2017. The Japanese Red Cross Hospital ICU, which was 12-bed closed mixed ICU, had approximately 800 ICU admissions per year during the study period. The ethics committee of the hospital approved this study and confirmed that the need for informed consent was waived due to the retrospective nature of the study.

### Study participants

We included patients aged ≥ 18 years who met the diagnostic criteria for sepsis based on the Sepsis-3 criteria at the time of the ICU admission and stayed in the ICU for ≥ 48 h [24–26]. The eligibility of patients who were admitted to the ICU were retrospectively evaluated by two of the authors prior to applying the Sepsis-3 criteria [27]. We excluded the following patients, because they were thought to have limited capacity to ambulate during their ICU stays: patients with acute cerebrovascular disease, progressive neuromuscular disease, post-cardiac arrest syndrome, unstable pelvic fracture, spinal injury with fracture of the spine, or multiple absent limbs. If a patient was readmitted to the ICU after discharge from the hospital during the study period, only data from the first admission was used for the analysis. All patients received the standard treatment based on surviving sepsis campaign guidelines 2012 [28] and 2016 [29].

### The Maebashi early mobilization protocol

In this study, mobilization was defined as rehabilitation at the level of sitting on the edge of the bed or more (e.g., standing beside or walking around the bed). At Maebashi Red Cross Hospital, there was no standardized protocol for the introduction of mobilization for patients admitted to the ICU, but in June 2015, the Maebashi early mobilization protocol was created. Details of the Maebashi early mobilization protocol are provided in Additional file 1. Although the Maebashi early mobilization protocol changed the timing of mobilization introduction, it did not change the mobilization level that was provided to the patients and the 20-min duration of mobilization per session. The discontinuation criterion at each rehabilitation session was described in Additional file 1, in line with recent expert consensus [30].

### Data collection

The data were retrospectively collected from electronic-based medical records [24]. We collected the following patient demographics and characteristics: age, sex, body mass index (BMI), Charlson Comorbidity Index (CCI), APACHE II and SOFA score at ICU admission, the main source of infection, the route to the ICU (e.g., emergency room, general ward), the ambulatory dependence before hospital admission, the diagnosis of septic shock at ICU admission, and the receipt of the Maebashi early mobilization protocol. We also collected data on when patients first received rehabilitation interventions and when patients first achieved mobilization during their ICU stays. In addition, we collected the data on the treatment patients received during their ICU stays: the use of the medical devices (invasive mechanical ventilator, extracorporeal membrane oxygenation [ECMO], and renal replacement therapy), corticosteroids, neuromuscular blockade, continuous analgesia with fentanyl, continuous sedation with benzodiazepines, propofol, or dexmedetomidine, and continuous vasopressor infusion (norepinephrine, dopamine, dobutamine, epinephrine, or vasopressin). For continuous analgesia, sedation, and vasopressor use, the details of the name of the drug used, its duration, and the average doses were also collected.

### Study outcomes

The primary outcomes were in-hospital mortality and ambulatory dependence at hospital discharge. The secondary outcomes were the lengths of the ICU and hospital stays and the total hospital costs. The total costs were calculated based on the Diagnosis Procedure Combination/Per-Diem Payment System [31] and converted from Japanese yen to US dollars at an exchange rate of 114 yen/dollar.

### Statistical analysis

First, we defined early mobilization (EM) as achieving mobilization within the first 3 days of ICU stay. Those who did not achieve mobilization during their ICU stay or achieved mobilization after the first 3 days were classified into the non-EM group. We compared the patient characteristics, treatments, and outcomes between the two groups by using the Mann–Whitney *U*-test and the Fisher’s exact test.

Second, we developed a multivariable logistic regression model to estimate a propensity score for each patient’s likelihood of achieving early mobilization. The covariates included to generate the propensity score were as follows: age, sex, BMI, CCI, APACHE II and total SOFA score at the ICU admission, the route to the ICU, ambulatory dependence before the hospital admission, the diagnosis of septic shock at ICU admission, the receipt of the Maebashi early mobilization protocol, and the treatments which patients received during their ICU stays [invasive mechanical ventilation, ECMO, renal replacement therapy, corticosteroid, neuromuscular blockade, analgesia with fentanyl, sedation with midazolam and propofol, and catecholamine use (noradrenaline, dopamine, or dobutamine)]. We then applied stabilized inverse probability weights (sIPWs) [32, 33] to calculate the adjusted odds ratios (OR) and 95% confidence intervals (CIs) of patients in the EM group relative to the non-EM group for the primary outcomes, and the adjusted means and 95% CIs for both groups for the secondary outcomes.

Next, we further analyzed data using alternative definitions of EM by changing the cutoff in days to mobilization by 1-day increments from 2 to 7 days. For each definition of EM, we implemented applied sIPWs as aforementioned to examine the changes in outcomes.

In addition, we performed two subgroup analyses: (i) excluding patients who did not achieve mobilization during their ICU stay from the non-EM group and (ii) excluding patients before June 2015, when the Maebashi early mobilization protocol was introduced. In each analysis, similar to the main analysis, we changed the cutoff in days to mobilization by 1-day increments from 2 to 7 days and applied sIPWs as aforementioned.

All analyses were conducted using Python version 3.8.12. A *P*-value of < 0.05 was considered statistically significant.

## Results

### Patient baseline characteristics and treatments

**Fig. 1:**
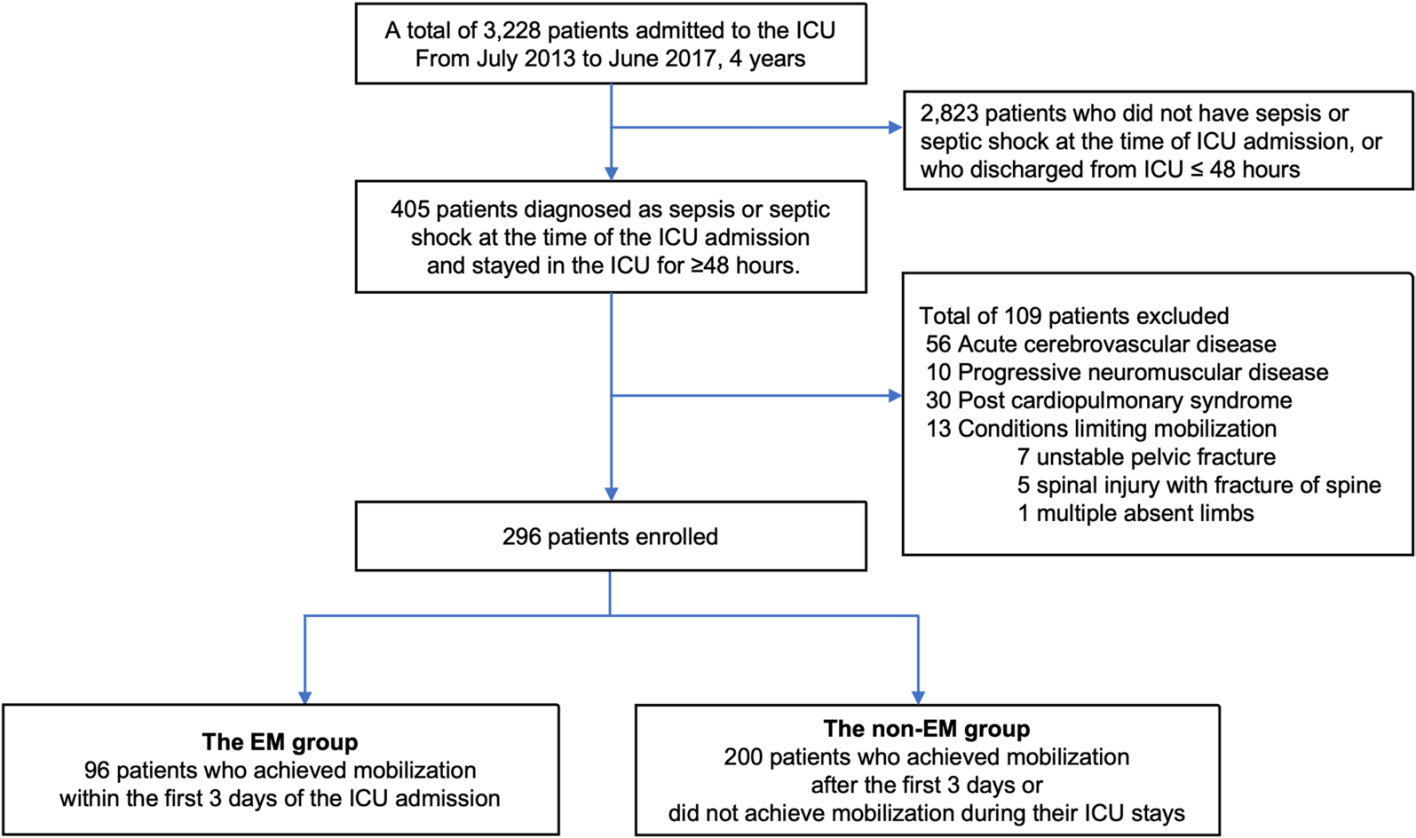
Flow chart of patient selection. *EM* early mobilization, *ICU* intensive care unit

The study flow of patient selection is shown in Fig. 1. Of 3228 ICU admissions during the study period, we included a total of 296 patients (9%) and classified 96 patients into the EM group and 200 patients into the non-EM group (Table 1).

**Table 1:** Demographics of all patients, patients in the EM group, and the non-EM group

Variable	All patients (*n* = 296)	Patients in the EM group (*n* = 96)	Patients in the non-EM group (*n* = 200)	*P*-value
Age (year), median [IQR]	75 [65–81]	74 [65–81]	75 [65–81]	0.90
Male sex, *n* (%)	200 (68%)	71 (74%)	129 (65%)	0.11
BMI (kg/m^2^), median [IQR]	21 [18–24]	22 [19–25]	21 [18–24]	0.13
Charlson Comorbidity Index, median [IQR]	2 [1–3]	2 [1–3]	2 [1–3]	0.39
APACHE II at ICU admission, median [IQR]	23 [19–28]	22 [18–27]	24 [20–28]	0.07
SOFA score at ICU admission, median [IQR]				
Total	8 [5–11]	7 [5–11]	9 [6–11]	0.09
Respiratory	2 [1–3]	2 [1–3]	2 [1–3]	0.35
Cardiovascular	3 [0–4]	3 [0–4]	4 [0–4]	0.01
Liver	0 [0–1]	0 [0–0]	0 [0–1]	0.43
Kidney	1 [0–2]	0 [0–2]	1 [0–2]	0.46
Coagulation	1 [0–2]	1 [0–2]	1 [0–2]	0.53
Nervous system	1 [0–2]	1 [0–2]	1 [0–2]	0.37
Main source of the infection, *n* (%)				
Abdomen	132 (45%)	41 (43%)	91 (46%)	0.71
Respiratory tract	94 (32%)	37 (39%)	57 (29%)	0.09
Urinary tract	31 (10%)	8 (8%)	23 (12%)	0.54
Soft tissue infection	24 (8%)	5 (5%)	19 (10%)	0.26
Others or unknown	15 (5%)	6 (6%)	10 (5%)	0.99
Admission to the ICU directly from the ED, *n* (%)	233 (79%)	75 (78%)	161 (79%)	0.88
Ambulatory dependence before the hospital admission, *n* (%)	48 (16%)	11 (11%)	37 (19%)	0.13
Septic shock at ICU admission, *n* (%)	190 (64%)	53 (55%)	137 (69%)	0.03
Patients who received the Maebashi early mobilization protocol, *n* (%)	138 (47%)	92 (96%)	46 (23%)	< 0.01
First intervention day for patients who received rehabilitation intervention during ICU stay (day), median [IQR]	1.8 [1.0–3.0]	1.0 [0.8–1.8]	2.9 [1.7–4.0]	< 0.01
First mobilization day for patients who achieved mobilization during ICU stay (day), median [IQR]	2.8 [1.7–5.1]	1.9 [1.3–2.4]	6.0 [4.5–9.0]	< 0.01

Patients who achieved mobilization within the first 3 days of the ICU admission were included in the EM group, while patients who did not achieve mobilization during their ICU stays or achieved mobilization after the first 3 days were included in the non-EM group. Of the patients in the non-EM group, 128 (64%) received rehabilitation therapy during their stay in the ICU, and 61 (31%) achieved mobilization during their stay in the ICU.

*APACHE* Acute Physiology and Chronic Health Evaluation, *BMI* Body Mass Index, *ED* emergency department, *EM* early mobilization, *ICU* intensive care unit, *IQR* interquartile range, *SOFA* Sequential Organ Failure Assessment

Patients in the EM group were more likely to be male (74% vs. 65%), have higher BMI (median: 22 vs. 21), and have lower APATCHE II and total SOFA scores at ICU admission (median APACHE II score, 22 vs. 24: median total SOFA score, 7 vs. 9) compared to patients in the non-EM group. Patients in the EM group were likely to have a respiratory tract infection (39% vs. 29%). In addition, patients in the EM group were less likely to be ambulatory dependent before hospital admission (11% vs. 19%), and less likely to have met the septic shock criteria at ICU admission (55% vs. 69%).

As for the treatments which patients received during their ICU stays (Table 2), patients in the EM group were less likely to have received invasive mechanical ventilation (61% vs. 70%). In addition, patients in the EM group were less likely to have received sedation with benzodiazepines during their ICU stays (21% vs. 37%) and catecholamines norepinephrine (69% vs. 78%), dopamine (27% vs. 43%), or dobutamine (9% vs. 21%).

**Table 2:** The details of treatments provided

Treatments	All patients (*n* = 296)	Patients in the EM group (*n* = 96)	Patients in the non-EM group (*n* = 200)	*P*-value
Management of respiratory and circulatory dynamics				
Invasive mechanical ventilation, *n* (%)	199 (67%)	59 (61%)	140 (70%)	0.15
ECMO, *n* (%)	17 (6%)	4 (4%)	13 (7%)	0.59
VA-ECMO	6 (2%)	0 (0%)	6 (3%)	0.18
VV-ECMO	11 (4%)	4 (4%)	7 (4%)	0.75
Renal dialysis, *n* (%)	94 (32%)	26 (27%)	68 (34%)	0.29
Medication treatment				
Corticosteroid, *n* (%)	71 (24%)	20 (21%)	50 (25%)	0.47
Neuromuscular blocking agent, *n* (%)	8 (3%)	1 (1%)	7 (4%)	0.44
Analgesia and sedation				
Continuous analgesia (fentanyl), *n* (%)	204 (69%)	63 (66%)	141 (71%)	0.35
Fentanyl duration (day), median [IQR]	2.6 [1.4–4.8]	2.0 [1.3–3.2]	3.5 [1.6–5.7]	0.05
Mean fentanyl dose (µg/h), median [IQR]	25.0 [21.0–36.2]	25.8 [21.3–39.2]	25.0 [20.9–35.1]	0.71
Continuous sedation, *n* (%)	201 (68%)	61 (63%)	140 (70.0%)	0.23
Total sedation duration (day), median [IQR]	2.4 [1.3–4.7]	1.7 [1.1–2.6]	2.7 [1.4–6.3]	0.04
Use of benzodiazepines, *n* (%)	93 (31%)	20 (21%)	73 (37%)	< 0.01
Mean benzodiazepine dose (mg/h), median [IQR]	4.4 [2.7–5.4]	5.0 [3.1–5.7]	4.0 [2.6–5.2]	< 0.01
Use of propofol, *n* (%)	136 (46%)	42 (44%)	94 (47%)	0.62
Mean propofol dose (mg/h), median [IQR]	50.7 [39.5–68.4]	50.9 [37.4–78.2]	49.4 [40.0–66.8]	0.50
Use of dexmedetomidine, *n* (%)	145 (49%)	43 (45%)	102 (51%)	0.32
Mean dexmedetomidine dose (µg/h), median [IQR]	16.2 [12.0–22.8]	18.0 [13.0–22.9]	16.0 [12.0–21.2]	0.70
Vasopressor				
Continuous vasopressor, *n* (%)	230 (78%)	70 (73%)	160 (80%)	0.18
Use of norepinephrine, *n* (%)	221 (75%)	66 (69%)	155 (78%)	0.12
Mean norepinephrine dose (10^–1^ µg/kg/min), median [IQR]	1.5 [1.0–2.2]	1.5 [0.8–2.1]	1.5 [1.1–2.4]	0.89
Use of dopamine, *n* (%)	112 (38%)	26 (27%)	86 (43%)	0.01
Mean dopamine dose (µg/kg/min), median [IQR]	4.0 [2.9–5.3]	4.0 [2.9–4.4]	4.0 [3.0–5.5]	< 0.01
Use of dobutamine, *n* (%)	50 (17%)	9 (9%)	41 (21%)	0.02
Mean dobutamine dose (µg/kg/min), median [IQR]	3.2 [2.3–4.7]	2.3 [2.0–2.7]	3.7 [2.6–5.2]	< 0.01
Use of epinepheline, *n* (%)	16 (5%)	3 (3%)	13 (7%)	0.28
Mean epinepheline dose (10^–1^ µg/kg/min), median [IQR]	1.1 [0.9–1.3]	0.9 [0.9–1.0]	1.2 [1.0–1.4]	< 0.01
Use of vasopressin, *n* (%)	51 (17%)	12 (13%)	39 (20%)	0.14
Mean vasopressin dose (units/h), median [IQR]	1.2 [1.0–1.7]	1.5 [1.2–1.7]	1.0 [0.9–1.7]	< 0.01

*ECMO* extracorporeal membrane oxygenation, *EM* early mobilization, *VA* venoarterial, *VV* venovenous

### Differences in outcomes between the EM and non-EM groups

As shown in Table 3, the in-hospital mortality of patients in the EM and non-EM groups were 7% vs. 24%, respectively, and the adjusted OR for the EM group compared to the non-EM group after applying sIPWs was 0.22 [95% CI 0.06–0.88]. The rates of ambulatory dependence at hospital discharge of patients in the EM and non-EM groups were 27% vs. 57%, and the adjusted OR for the EM group compared to the non-EM group after applying sIPWs was 0.24 [95% CI 0.09–0.61].

**Table 3:** Study outcomes of patients in the EM group vs. the non-EM group

Outcomes	Unadjusted outcomes				Adjusted outcomes	
	All patients (*n* = 296)	Patients in the EM group (*n* = 96)	Patients in the non-EM group (*n* = 200)	*P*-value	Patients in the EM group (*n* = 96)	Patients in the non-EM group (*n* = 200)
Primary outcomes	*n* (%)	*n* (%)	*n* (%)		Adjusted odds ratio [95% CI]	Reference
In-hospital mortality	55 (19%)	7 (7%)	48 (24%)	< 0.01	0.22 [95% CI 0.06–0.88]	–
Ambulatory dependence at the hospital discharge	139 (47%)	26 (27%)	113 (57%)	< 0.01	0.24 [95% CI 0.09–0.61]	–

Secondary outcomes	Median [IQR]	Median [IQR]	Median [IQR]		Adjusted mean value [95% CI]	Adjusted mean value [95% CI]
Length of the ICU stays (day)	6.1 [4.5–9.0]	5.3 [4.2–6.8]	6.5 [5.0–10.7]	< 0.01	5.8 [4.2–7.4]	9.0 [7.9–10.0]
Length of the hospital stays (day)	33.4 [18.2–53.1]	28.3 [16.8–46.1]	34.0 [19.5–61.1]	0.10	36.6 [31.6–41.7]	44.3 [37.1–51.5]
Total hospital costs (US dollars)	27,954 [17,902–50,058]	24,823 [14,778–39,703]	32,515 [20,060–51,854]	< 0.01	28,351 [22,267–34, 36]	37,740 [32888–42952]

Adjusted outcomes means the outcomes adjusted with the sIPWs using the following covariates to generate the propensity score: age, sex, BMI, CCI, APACHE II and total SOFA score at ICU admission, the main source of the infection, the route to the ICU, the ambulatory dependence before the hospital admission, the diagnosis of septic shock on the ICU admission, and the receipt of the Maebashi early mobilization protocol and the treatments which patients received during their ICU stays (invasive mechanical ventilation, ECMO, renal replacement therapy, steroid, neuromuscular blockade, analgesia with fentanyl, sedation with midazolam and propofol, and receipt of catecholamines noradrenaline, dopamine, or dobutamine). Unadjusted outcomes were compared using the Mann–Whitney *U*-test and Fisher’s exact test

*CI* confidence interval, *EM* early mobilization, *ICU* intensive care unit, *OR* odds ratio, *sIPWs* stabilized inverse probability weightings

Analyses of the secondary outcomes for the two groups are shown in Table 3. The mean difference of the outcomes (percentage points, %) between the EM group and the non-EM group estimated after applying sIPWs were as follows: length of the ICU stay, − 3.2 days (− 36%); length of the hospital stay, − 7.7 days (− 14%); the total hospital costs, − 9389 dollars (− 25%).

### Changes in the patient outcomes after shifting the cutoff days of EM

**Fig. 2:**
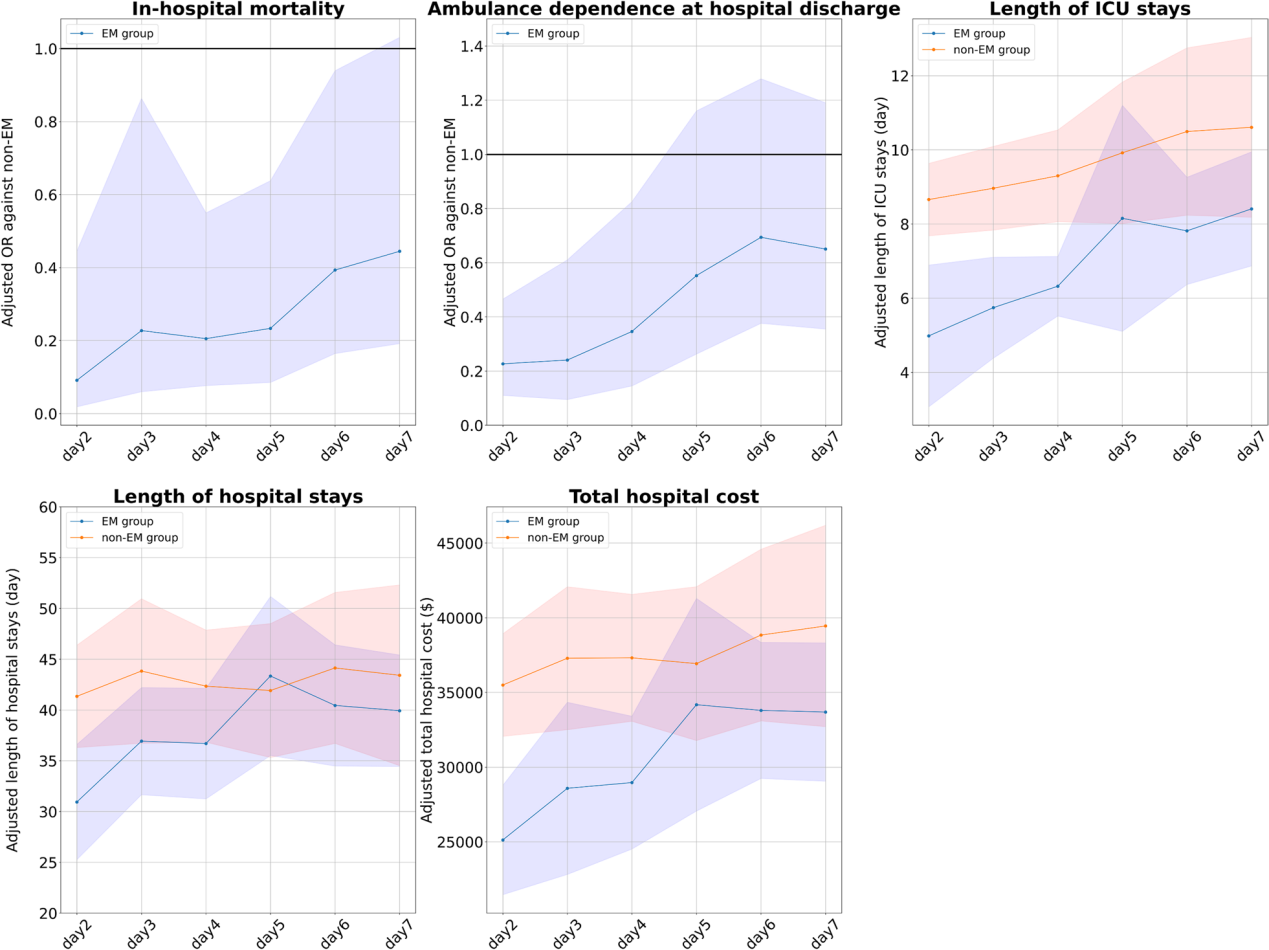
Outcomes of patients in each group when the cutoff for early mobilization is changed. The graphs show the adjusted OR or mean difference and 95% CIs of the EM group (blue) and the non-EM (red) group for each outcome. The horizontal axis of the graph shows the cutoff day for EM. (e.g., for cutoff of day 2, the EM group includes patients who achieved mobilization within the first 2 days of ICU admission.). *CI* confidence interval, *EM* early mobilization, *ICU* intensive care unit, *OR* odds ratio

Figure 2 shows the outcomes for the EM and non-EM groups when the definition of the EM was shifted by 1-day increments from 2 to 7 days. In this analysis, the adjusted OR of the in-hospital mortality and the ambulatory dependence at hospital discharge in the EM group tended to be larger when the cutoff of EM was set at 2–4 days (e.g., adjusted OR of the in-hospital mortality in the EM group vs. the non-EM group when EM was defined at day 2 of ICU stay was 0.21 [95% CI 0.07–0.61], while when cutoff for EM was set at day 7 of ICU stay was 0.45 [95% CI 0.20–1.04]). As for the secondary outcomes, when EM was defined at days 2–4, the difference in outcomes between the EM and non-EM groups tended to be greater.

### Subgroup analysis (i): excluding patients who did not achieve mobilization during their ICU stays

**Fig. 3:**
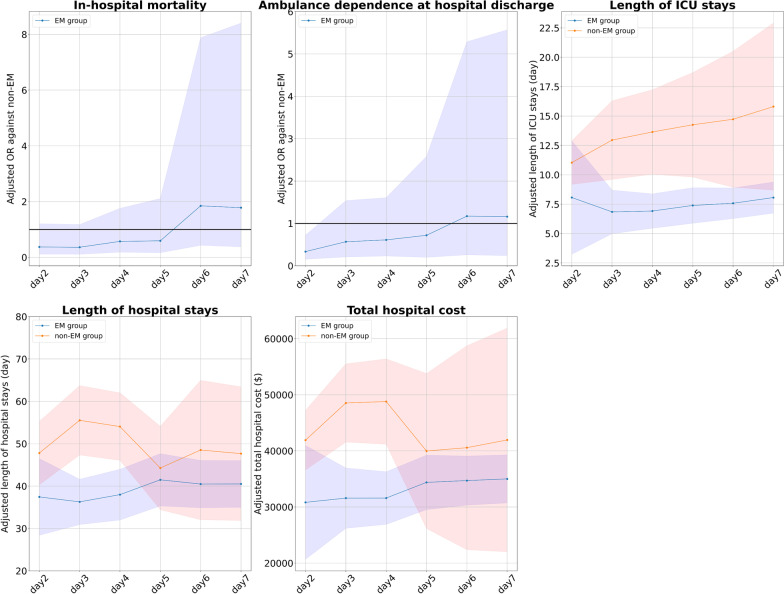
Outcomes for patients in each group when patients who did not achieve EM are excluded. The graphs show the adjusted OR or mean difference and 95% CIs of the EM group (blue) and the non-EM (red) group for each outcome. The horizontal axis of the graph shows the cutoff day for EM. (e.g., for cutoff of day 2, the EM group includes patients who achieved mobilization within the first 2 days of ICU admission.). *CI* confidence interval, *EM* early mobilization, *ICU* intensive care unit, *OR* odds ratio

Out of the total 296 patients, there were 139 patients (47%) who did not achieve mobilization during their ICU stays. We excluded these 139 patients and shifted the definition of the EM in 1-day increments from days 2 to 7. We then applied sIPWs as aforementioned. As shown in Fig. 3, we found that the results of the subgroup analysis were consistent with the main analysis.

### Subgroup analysis (ii): excluding patients before the introduction of the early mobilization protocol

**Fig. 4:**
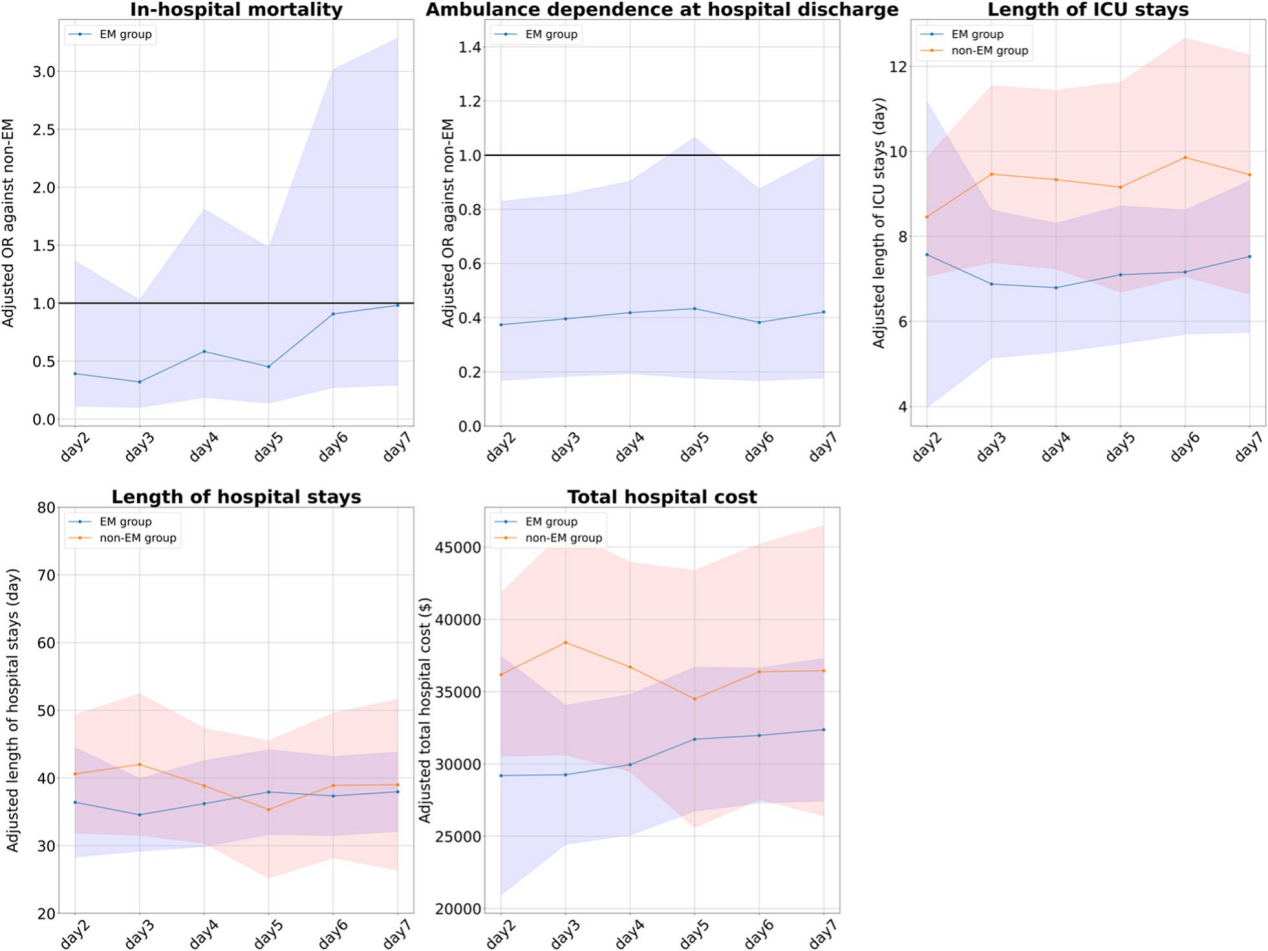
Outcomes for patients in each group, excluding patients before the introduction of the Maebashi early mobilization protocol are excluded. The graphs show the adjusted OR or mean difference and 95% CIs of the EM group (blue) and the non-EM (red) group for each outcome. The horizontal axis of the graph shows the cutoff day for EM. (e.g., for cutoff of day 2, the EM group includes patients who achieved mobilization within the first 2 days of ICU admission.). *CI* confidence interval, *EM* early mobilization, *ICU* intensive care unit, *OR* odds ratio

Out of a total of 296 patients, there were 158 patients (53%) before the Maebashi early mobilization protocol was introduced. We excluded these 158 patients, and of the remaining 138 (47%), we identified 87 in the EM group and 51 in the non-EM group. Then, we performed the same analysis as the primary analysis. As shown in Fig. 4, we found that the results of this subgroup analysis were also consistent with the main analysis.

## Discussion

In this retrospective study of 296 adult patients who were admitted to the ICU with sepsis, we found that patients who achieved mobilization within the first 3 days of the ICU admission had better outcomes than those who did not achieve mobilization during the ICU stay or achieved mobilization after the first 3 days. When we changed the cutoff for EM in 1-day increments from days 2 to 7, EM group patients had better outcomes than non-EM group patients when the cutoff was set at days 2 to 4 than days 5 to 7. The results were consistent with the main analyses after excluding patients who did not achieve mobilization during their ICU stays and after excluding patients before the introduction of the Maebashi early mobilization protocol.

Previous studies have shown that early mobilization (e.g., within 3 days) improves outcomes of ICU patients [14–17], while late mobilization (e.g., within 7 days) does not substantially improve outcomes [22]. This is consistent with our findings, which underscore the crucial principle that there should be a time threshold for patient recovery depending on when mobilization practice is introduced for ICU patients.

There are two possible explanations for why early mobilization may improve patient outcomes. First, early mobilization prevents the progression of muscle atrophy. It has been suggested that in patients with severe inflammatory diseases such as sepsis, muscle necrosis due to the inflammatory response, combined with the patient’s immobility, leads to rapid progression of muscle atrophy from an early phase of the ICU stay [34–37]. Indeed, in a recent prospective study of critically ill patients admitted to the ICU, 54% of the patients with thigh muscle weakness had myonecrosis [38]. Thus, muscle atrophy that occurs early in the ICU stay could be considered an organ failure that requires early intervention. Therefore, introducing mobilization to patients with sepsis early in their ICU stays may result in the prevention of muscle atrophy development and progression.

Second, in the late stages of inflammatory disease, patients experience increased protein catabolism [39, 40]. It has been shown that the pathology of the late phase of inflammatory diseases differs from that of the early phase [41, 42]. Vanzant et al. explain that the early phase of inflammatory diseases is dominated by systemic inflammatory response syndrome (SIRS), while the late phase is dominated by compensatory anti-inflammatory response syndrome (CARS), and patients with CARS tend to have cachexia due to excessive protein catabolism [42]. Our study implies that patients who achieved mobilization late (days 5–7) after admission to the ICU may experience less improvements in the outcome. This could be attributed to the increased protein catabolism accelerated by mobilization therapy for patients in the later stages of inflammatory disease. In such patients, not only early mobilization practice but also nutritional support tailored to the patient’s nutritional status could have improved outcomes, which should be further investigated in future studies.

The Surviving Sepsis Campaign Guidelines 2021 for adult septic patients has been recently updated to incorporate various treatment strategies to improve the long-term prognosis of septic patients, including rehabilitation plans and financial and social support involving the patient’s family [43]. However, these strategies focus primarily on the care patients receive after they are discharged from the ICU, and the mobilization of patients from during the ICU stay has not been sufficiently discussed. Although there are various barriers against mobilization, especially in the early phases of ICU stay (e.g., hemodynamic instability, altered level of consciousness, etc.), the current literature, including cohort studies and RCTs, supports the feasibility of achieving mobilization within the first 3 days of ICU stay or even the first 2 days [26, 30]. Our study sends an important message to all clinicians involved in ICU care that mobilization strategies for patients with sepsis should be optimized even in the early phase of their ICU stays, such as the first 2–4 days, to not miss the opportunity for adequate recovery from critical illness.

## Potential limitations

First, in June 2015 (during the study period), a new protocol for mobilization during ICU stays was introduced at Maebashi Red Cross Hospital. This facilitated introducing early mobilization, which could be a potential source of bias. However, there was no change in the level of mobilization therapy or duration of therapy per session, so including patients before the introduction of the protocol should not be a major problem in estimating the effect of mobilization therapy on patient outcomes. In addition, we added a binary variable of whether the patient received the new protocol as a covariate in the calculation of the propensity score, which allowed us to adjust for potential bias. Second, we identified septic patients based on the Sepsis-3 criteria [27], which was introduced in 2016. Therefore, patients to be included in the study prior to 2016 were identified retrospectively by the authors, which could have resulted in misclassification. To our best, we have minimized this problem by diagnosing patients as septic when two authors’ decisions were the same. However, the announcement of major guidelines published during the study period (e.g., the surviving sepsis campaign guideline 2016 [29], the clinical practice guidelines for pain, agitation/sedation, and delirium 2013 [44]) could have acted as potential confounders that could not be fully adjusted. Third, because we were unable to obtain a detailed time series of treatment information during the ICU stays, we may not have accurately adjusted for the effect of treatments. Although we adjusted for binary variables related to treatment during the ICU stays, it would be desirable to obtain detailed time-series information on treatment and adjust for them in future studies. Finally, our findings may have limited generalizability because our study was conducted retrospectively at a single center and the number of patients is not large. To verify our findings, larger multi-center prospective studies are warranted.

## Conclusions

For patients admitted to the ICU with sepsis, achieving mobilization within the first 3 days of ICU stay was significantly associated with better outcomes. The first 2–4 days might be the optimal target to achieve mobilization, but this needs to be validated in further studies.

## Supplementary Information

**Additional file 1.** The differences between the mobilization strategy before and after the initiation of the Maebashi early mobilization protocol.

## Data Availability

The datasets used and/or analyzed during the current study are available from the corresponding author on reasonable request.

## Abbreviations

ICU: Intensive care unit
PICS: Post-intensive care syndrome
RCTs: Randomized controlled trials
EM: Early mobilization
sIPW: Stabilized inverse probability weighting
OR: Odds ratios
CI: Confidence intervals
BMI: Body mass index
CCI: Charlson Comorbidity Index
APACHE: Acute Physiology and Chronic Health Evaluation
SOFA: Sequential Organ Failure Assessment
ECMO: Extracorporeal membrane oxygenation
SIRS: Systemic inflammatory response syndrome
CIRS: Compensatory anti-inflammatory response syndrome

## References

[1] Kim T, Huh S, Kim SY (2019). ICU rehabilitation is associated with reduced long-term mortality from sepsis in patients with low skeletal muscle mass: a case control study. Ann Transl Med.

[2] Verceles AC, Wells CL, Sorkin JD (2018). A multimodal rehabilitation program for patients with ICU acquired weakness improves ventilator weaning and discharge home. J Crit Care.

[3] Anekwe DE, Biswas S, Bussières A, Spahija J (2020). Early rehabilitation reduces the likelihood of developing intensive care unit-acquired weakness: a systematic review and meta-analysis. Physiotherapy.

[4] Rawal G, Yadav S, Kumar R (2017). Post-intensive care syndrome: an overview. J Transl Intern Med.

[5] Parker A, Sricharoenchai T, Needham DM (2013). Early rehabilitation in the intensive care unit: preventing physical and mental health impairments. Curr Phys Med Rehabil Rep.

[6] Inoue S, Hatakeyama J, Kondo Y (2019). Post-intensive care syndrome: its pathophysiology, prevention, and future directions. Acute Med Surg.

[7] Colbenson GA, Johnson A, Wilson ME (2019). Post-intensive care syndrome: impact, prevention, and management. Breathe (Sheff).

[8] Hermans G, Van Mechelen H, Clerckx B (2014). Acute outcomes and 1-year mortality of intensive care unit-acquired weakness. A cohort study and propensity-matched analysis. Am J Respir Crit Care Med.

[9] Dinglas VD, Aronson Friedman L, Colantuoni E (2017). Muscle weakness and 5-year survival in acute respiratory distress syndrome survivors. Crit Care Med.

[10] Herridge MS, Batt J, Dos SC (2014). ICU-acquired weakness, morbidity, and death. Am J Respir Crit Care Med.

[11] Harvey MA, Davidson JE (2016). Postintensive care syndrome: right care, right now…and later. Crit Care Med.

[12] Hill AD, Fowler RA, Pinto R, Herridge MS, Cuthbertson BH, Scales DC (2016). Long-term outcomes and healthcare utilization following critical illness—a population-based study. Crit Care.

[13] Held N, Moss M (2019). Optimizing post-intensive care unit rehabilitation. Turk Thorac J.

[14] Sawada Y, Sasabuchi Y, Nakahara Y (2018). Early rehabilitation and in-hospital mortality in intensive care patients with community-acquired pneumonia. Am J Crit Care.

[15] Bailey P, Thomsen GE, Spuhler VJ (2007). Early activity is feasible and safe in respiratory failure patients. Crit Care Med.

[16] Ding N, Zhang Z, Zhang C (2019). What is the optimum time for initiation of early mobilization in mechanically ventilated patients? A network meta-analysis. PLoS ONE.

[17] Morris PE, Griffin L, Berry M (2011). Receiving early mobility during an intensive care unit admission is a predictor of improved outcomes in acute respiratory failure. Am J Med Sci.

[18] Fuke R, Hifumi T, Kondo Y (2018). Early rehabilitation to prevent postintensive care syndrome in patients with critical illness: a systematic review and meta-analysis. BMJ Open.

[19] Hashem MD, Nelliot A, Needham DM (2016). Early mobilization and rehabilitation in the ICU: moving back to the future. Respir Care.

[20] Kondo Y, Fuke R, Hifumi T (2017). Early rehabilitation for the prevention of postintensive care syndrome in critically ill patients: a study protocol for a systematic review and meta-analysis. BMJ Open.

[21] Titsworth WL, Hester J, Correia T (2012). The effect of increased mobility on morbidity in the neurointensive care unit. J Neurosurg.

[22] Okada Y, Unoki T, Matsuishi Y, Egawa Y, Hayashida K, Inoue S (2019). Early versus delayed mobilization for in-hospital mortality and health-related quality of life among critically ill patients: a systematic review and meta-analysis. J Intensive Care.

[23] Iwashyna TJ, Ely EW, Smith DM, Langa KM (2010). Long-term cognitive impairment and functional disability among survivors of severe sepsis. JAMA.

[24] Liu K, Ogura T, Takahashi K (2019). A progressive early mobilization program is significantly associated with clinical and economic improvement: a single-center quality comparison study. Crit Care Med.

[25] Watanabe S, Morita Y, Suzuki S (2021). Effects of the intensity and activity time of early rehabilitation on activities of daily living dependence in mechanically ventilated patients. Prog Rehabil Med.

[26] Watanabe S, Liu K, Morita Y (2021). Changes in barriers to implementing early mobilization in the intensive care unit: a single center retrospective cohort study. Nagoya J Med Sci.

[27] Singer M, Deutschman CS, Seymour CW (2016). The third international consensus definitions for sepsis and septic shock (Sepsis-3). JAMA.

[28] Dellinger RP, Levy MM, Rhodes A (2013). Surviving sepsis campaign: international guidelines for management of severe sepsis and septic shock: 2012. Crit Care Med.

[29] Rhodes A, Evans LE, Alhazzani W (2017). Surviving sepsis campaign: international guidelines for management of sepsis and septic shock: 2016. Intensive Care Med.

[30] Hodgson CL, Stiller K, Needham DM (2014). Expert consensus and recommendations on safety criteria for active mobilization of mechanically ventilated critically ill adults. Crit Care.

[31] Hayashida K, Murakami G, Matsuda S, Fushimi K (2021). History and profile of diagnosis procedure combination (DPC): development of a real data collection system for acute inpatient care in Japan. J Epidemiol.

[32] Ali MS, Prieto-Alhambra D, Lopes LC (2019). Propensity score methods in health technology assessment: principles, extended applications, and recent advances. Front Pharmacol.

[33] Hernán MA, Robins JM (2020). Causal inference: what if.

[34] Kress JP, Hall JB (2014). ICU-acquired weakness and recovery from critical illness. N Engl J Med.

[35] Stäuble CG, Helming M, Martyn JAJ, Blobner M, Fink H (2016). Neuromuscular recovery is prolonged after immobilization or superimposition of inflammation with immobilization compared to inflammation alone: data from a preclinical model. Crit Care Med.

[36] Bonaldo P, Sandri M (2013). Cellular and molecular mechanisms of muscle atrophy. Dis Model Mech.

[37] Cohen S, Nathan JA, Goldberg AL (2015). Muscle wasting in disease: molecular mechanisms and promising therapies. Nat Rev Drug Discov.

[38] Puthucheary ZA, Rawal J, McPhail M (2013). Acute skeletal muscle wasting in critical illness. JAMA.

[39] Bouharras El Idrissi H, Molina López J, Pérez Moreno I (2015). Imbalances in protein metabolism in critical care patient with systemic inflammatory response syndrome at admission in intensive care unit. Nutr Hosp.

[40] Mercier S, Breuillé D, Mosoni L, Obled C, Patureau MP (2002). Chronic inflammation alters protein metabolism in several organs of adult rats. J Nutr.

[41] Ward NS, Casserly B, Ayala A (2008). The compensatory anti-inflammatory response syndrome (CARS) in critically ill patients. Clin Chest Med.

[42] Vanzant EL, Lopez CM, Ozrazgat-Baslanti T (2014). Persistent inflammation, immunosuppression, and catabolism syndrome after severe blunt trauma. J Trauma Acute Care Surg.

[43] Evans L, Rhodes A, Alhazzani W (2021). Surviving sepsis campaign: international guidelines for management of sepsis and septic shock 2021. Intensive Care Med.

[44] Barr J, Fraser GL, Puntillo K (2013). Clinical practice guidelines for the management of pain, agitation, and delirium in adult patients in the intensive care unit. Crit Care Med.

