# Supplementary material for: Optimal timing of introducing mobilization therapy for ICU patients with sepsis
Source: J Intensive Care. 2022 Apr 25;10:22. doi: 10.1186/s40560-022-00613-8 (PMC9036689; doi:10.1186/s40560-022-00613-8)
Supplement: Supplementary file 1 — Additional file 1. The differences between the mobilization strategy before and after the initiation of the Maebashi early mobilization protocol. [file 40560_2022_613_MOESM1_ESM.docx]

**Additional file 1. The differences between the mobilization strategy before and after the initiation of the Maebashi early mobilization protocol**

| **Comparison items** | **Mobilization strategy before the Maebashi EM protocol was introduced** | **Mobilization strategy after the Maebashi EM protocol was introduced** |
| --- | --- | --- |
| ICU staff: nurses | Nurse-to-patient ratio is 2, including one nurse certified in critical care. | Not changed |
| ICU staff: physicians | One intensivist, 3-4 fellows, one junior resident. Physician to patient ratio is 2. | Not changed |
| ICU staff: physical therapists | No dedicated physical therapists in the ICU. A physical therapist is assigned to a patient and become available for EM when a rehabilitation order is written. | Not changed |
| When is the rehabilitation ordered? | Written when the ICU physician determines that rehabilitation is necessary for the patient to recover his/her physical function. The physical therapist in charge decides when to start. | Automatically written for all ICU admissions. The rehabilitation will start as soon as the rehabilitation order is received at the Department of Rehabilitation |
| Who will decide the rehabilitation intensity provided to the patients? | The physical therapists in charge decide the content of rehabilitation according to the patient condition. | ICU physicians evaluate the medical condition including respiratory, circulatory, and level of consciousness and determine the rehabilitation level according to the condition of the patient. |
| What rehabilitation intensity level is supposed to be provided to the patients? | Level 1: No mobilization, bed exercise such as passive range of motion, passive transfer to chair  Level 2: sitting position in bed, including using a cycling ergometer and active range of motion  Level 3: sitting on the edge of the bed  Level 4: active transfer to chair  Level 5: standing, stepping in place, ambulating | Not changed |
| How often is the rehabilitation provided? | Depends on the physical therapists in charge. | All patients should receive at least one rehabilitation session each day. |
| How long is the rehabilitation? | The duration of a session should be 20 minutes at most. | Not changed |
| Who are the providers of the rehabilitation sessions? | Primarily the physical therapist, occasionally the ICU physician or nurse. | Multidisciplinary team consisting of an ICU physician, a nurse, and a physical therapist. If a physical therapist is not available, an ICU physician and nurses provides rehabilitation. |
| Who decides to stop the rehabilitation and how? | There were no clearly defined criteria, but the physical therapists followed the same criteria as after the protocol was implemented. | The ICU physician decided whether to continue or discontinue the session based on the patient’s medical condition or discontinuance criteria as follows:  1) a fall to the knees or to the ground, 2) tachycardia (>130/min) or bradycardia(<40/min), 3) hypertension (systolic blood pressure >180 mmHg), 4) hypotension (systolic blood pressure <80 mmHg), 5) symptomatic orthostatic hypotension, 6) arrhythmias except for a pre-existing arrhythmia, 7) myocardial infarction-associated symptoms, 8) desaturation (peripheral capillary oxygen saturation <88%), 9) abnormal respiratory rate (>40/min or <5/min), 10) asynchrony with mechanical ventilation, 11) patient’s intolerance to request to stop rehabilitation, 12) cardiopulmonary arrest, 13) bleeding, 14) unexpected/inadvertent removal of medical devices (an endotracheal tube, feeding tube, chest tube, abdominal drain, urinary catheter, arterial catheter, peripheral or central venous catheter, or hemodialysis catheter.) |

1. When physical therapists want to mobilize a patient at an active rehabilitation level, such as sitting on edge of bed, standing, or ambulating, they could request help to the nurse or the ICU physician, but whether they could attend depends on the burden of their work on that day
2. The available ICU physicians are one of five ICU physicians in the ICU that day and not a resource from outside the ICU.

Abbreviations: ECMO: extracorporeal membrane oxygenation, EM: early mobilization, ICU: intensive care unit, RASS: Richmond agitation sedation scale
